# Voluntary Inhibition of Physiological Mirror Activity: An EEG-EMG Study

**DOI:** 10.1523/ENEURO.0326-20.2020

**Published:** 2020-10-27

**Authors:** T. Maudrich, R. Kenville, D. Maudrich, A. Villringer, P. Ragert, V. V. Nikulin

**Affiliations:** 1 Department of Neurology, Max Planck Institute for Human Cognitive and Brain Sciences, Leipzig D-04103, Germany; 2 Institute for General Kinesiology and Exercise Science, Faculty of Sports Science, University of Leipzig, Leipzig D-04109, Germany; 3 MindBrainBody Institute at Berlin School of Mind and Brain, Charité-University Medicine Berlin, Berlin 10099, Germany; 4 Clinic for Cognitive Neurology, University Hospital Leipzig, Leipzig D-04103, Germany; 5 Centre for Cognition and Decision Making, Institute for Cognitive Neuroscience, National Research University Higher School of Economics, Moscow 101000, Russian Federation; 6 Neurophysics Group, Department of Neurology, Charité-University Medicine Berlin, Berlin 10117, Germany

**Keywords:** δ power, EEG, EMG, inhibition, physiological mirror activity

## Abstract

Physiological mirror activity (pMA), observed in healthy human adults, describes the involuntary co-activation of contralateral homologous muscles during unilateral limb movements. Here we provide novel evidence, using neuromuscular measurements (electromyography; EMG), that the amplitude of pMA can be voluntarily inhibited during unilateral isometric contractions of intrinsic hand muscles after informing human participants (10 male, 10 female) about its presence and establishing a basic understanding of pMA mechanisms through a standardized protocol. Importantly, significant suppression of pMA was observed immediately after participants were asked to inhibit it, despite the absence of any online feedback during task execution and without special training. Moreover, we observed that the decrease of pMA was specifically accompanied by an increase in relative frontal δ power recorded with electroencephalography (EEG). Correlation analysis further revealed an inverse association between the individual amplitude of pMA and frontal δ power that reached significance once participants started to inhibit. Taken together, these results suggest that δ power in frontal regions might reflect executive processes exerting inhibitory control over unintentional motor output, in this case pMA. Our results provide an initial reference point for the development of therapeutic applications related to the neurorehabilitation of involuntary movements which could be realized through the suppression of pMA observed in the elderly before it would fully manifest in undesirable overt movement patterns.

## Significance Statement

Unilateral movements evoke unintended activation of contralateral muscles, especially when such movements are performed with high effort. This motor phenomenon, termed mirror activity (MA), is observed not only in pathologic conditions of the CNS but also in healthy individuals, where it is known as physiological MA (pMA). Surprisingly, as we show in our study, participants are capable of suppressing this unintended co-activation after being informed of its presence. Suppression was accompanied by increased δ power in frontal areas. This suggests that unintentional motor output can be suppressed via executive processes, i.e., inhibitory control. We offer an initial reference point for therapeutic applications regarding neurorehabilitation of involuntary movements and a possible strategy to counteract the age-related increase in MA.

## Introduction

Humans rely heavily on the bilateral independence of the limbs. A critical phenomenon that can impair such independence is termed mirror activity (MA) or associated movements (Post et al., 2009). MA involves involuntary muscle activity in the contralateral homolog of the purposely activated muscle. In severe cases, known as pathologic mirror movements (Galléa et al., 2011), this involuntary co-activation of the contralateral limb leads to overt movement that can impair even simple unilateral tasks like reaching and finger tapping. However, even in neurologically healthy adults, physiological MA (pMA) has been observed in homologous muscles (Cincotta and Ziemann, 2008). This phenomenon is hypothesized to occur because of a spillover of the initially lateralized motor command from the contralateral (controlling the active limb) toward the ipsilateral hemisphere (controlling the inactive limb), a mechanism termed motor overflow (Yensen, 1965; Perez and Cohen, 2008). Crossed facilitation within the human motor cortex, i.e., bilateral variation in the excitability of corticospinal projections, is generally observed during intentionally unilateral actions (Carson, 2005). The resulting pMA depends on the functional requirements of unilateral motor tasks (Cincotta and Ziemann, 2008). This is especially true for the level of neural drive toward the muscles engaged in the voluntary movement (Todor and Lazarus, 1986), i.e., the strength and duration of contractions (van Duinen et al., 2008; Sehm et al., 2010). Furthermore, it has been proposed that the occurrence and extent of pMA follows a U-shaped distribution across the life-span (Koerte et al., 2010). Typically, stronger pMA is observed in young children and rapidly decreases as the child enters adolescence (Mayston et al., 1999). In the third decade, pMA gradually reappears (Koerte et al., 2010). This course of pMA occurrence may reflect the processes of increasing inhibition during adolescence, with the later reappearance suggesting an age-related loss of central inhibition (Carr, 2010). Thus, it has been speculated that increased levels of pMA might be one of many altered patterns of muscle activation contributing to the decline in motor performance and manual dexterity that accompanies healthy aging (Shinohara et al., 2003).

Volitional control and suppression of involuntary movements have been demonstrated previously in patients with tremors, dyskinetic disorders, and Tourette syndrome (Koller and Biary, 1989). This suggests that focused attention enables inhibitory mechanisms capable of controlling unintentional motor output. Based on this assumption, we hypothesized that pMA would be present when participants are unaware of such a phenomenon but can be inhibited after attention has been shifted toward its presence. Indeed, one previous investigation was able to demonstrate, in young and older adults, inhibition of contralateral muscle activity during unilateral contractions with and without visual feedback during task execution (Addamo et al., 2010). The authors used measurements of force exerted by the mirror limb to quantify pMA. However, pMA does not always lead to observable involuntary movement or torque production, which renders force measurements insufficient to capture neurophysiological aspects of pMA in its entirety.

We aim here to provide evidence for voluntary pMA inhibition based on electromyographic (EMG) measurements, allowing for the quantification of even subtle muscular activity. We propose that a possible inhibition of pMA might be mediated through higher-order cognitive control (exerted by frontal cortical regions; Addamo et al., 2010), resulting in a suppression of motor-related brain areas and finally limiting involuntary motor output. To verify this, multichannel electroencephalography (EEG) was recorded to investigate spectral power changes associated with potential inhibitory processes. Based on previous studies investigating motor inhibitory processes (Kaiser et al., 2019) and executive processes involved in lateralized inhibition of symmetric contractions (Tisseyre et al., 2019), it was hypothesized that such voluntary inhibition would be associated with increases in δ (1–3 Hz) and θ (4–8 Hz) power. Evidence for successful inhibition of pMA and underlying neural mechanisms could provide an initial reference point for the development of therapeutic applications aimed at involuntary movement prevention. This approach could further provide a possible strategy to counteract or prevent the increase in pMA documented in the elderly.

## Materials and Methods

### Ethical approval

This study was supported by the local ethics committee of the University of Leipzig (ref.-nr. 423/18-ek). All participants gave written informed consent to participate in the experiment and were compensated for involvement, according to the Helsinki Declaration.

### Participants

Twenty neurologically healthy adults participated in the experiment (10 males, 10 females, age: 25.6 ± 5.4; mean ± SD). All participants were right-handed according to the Oldfield handedness inventory (Oldfield, 1971). Exclusion criteria consisted of neurologic pathologies, pregnancy, centrally active drug use, regular sport, or musical participation exceeding 2 h/week.

### Experimental design

Participants were seated comfortably in a chair and operated a custom-made force sensor with their thumb and index finger of one hand. The other hand rested on a pillow. Before starting the experiment, all participants performed a maximum voluntary contraction (MVC) test with both hands in the form of a pinch-contraction of the thumb and index finger. Three 5-s maximum contractions per hand were conducted, with 30 s of rest between each. The trial with the largest peak force was selected as the individual’s MVC. This was done to normalize EMG amplitudes during data processing and scale the required force level of the following experiment (40% MVC) to the individual strength capabilities. Subsequently, participants had to vertically move a cursor on a computer screen (placed in ∼1.5-m distance) repetitively into a target field by applying force to the sensor (set to require 40 ± 5% MVC) with their left hand while the right hand was resting in the prone position on the pillow. The target field was visible for 5 s and was followed by a 5-s rest period during which muscles should be completely relaxed. Continuous force data were recorded and used to (1) control that participants reached the target field and (2) compute the duration until participants reached the target field after it appeared on the computer screen for each contraction, i.e., ramp contraction time (RCT). The first 30 contractions served as a baseline block (BL), during which participants were naive concerning the study aim, i.e., at no time during BL were they aware of, or receive feedback about, ongoing pMA. However, pMA occurrence was confirmed through continuous observation of EMG signals during task execution. Following the BL, participants were thoroughly educated on the pMA phenomenon using standardized verbal instructions where their attention was directed toward involuntary muscle activity of their resting hand (Table 1). Afterward, participants were asked to actively inhibit the occurrence of pMA in subsequent force blocks by a self-chosen mental strategy. The goal was to minimize EMG amplitude of the non-performing hand without changing the motor performance of the left hand. Feedback about participants’ inhibition of pMA was provided verbally based on online monitoring by the researcher after the completion of each block. Online feedback was not provided. That means that participants neither received online EMG feedback nor verbal instructions during contractions. Participants could see both hands at all times but were instructed to fixate their gaze on the computer screen in front of them during all contractions. In addition to the BL, four more blocks of 30 contractions each were performed (I-block, II-block, III-block, and IV-block), resulting in a total of 150 epochs. In between blocks, a resting period of 3 min was granted.

**Table 1:** Standardized verbal instruction given during the experiment

Timepoint	Instruction
After baseline (BL) block	The goal of this study is to investigate the so-called mirror activity phenomenon. That means that every time you perform a contraction with one hand, we can also measure a weak activity in the contralateral resting hand. From now on, please try to suppress this involuntary activity as much as possible by a self-chosen strategy. However, keep the performance of the left hand identical.
After each consecutive block	Good job. However, we still see some involuntary muscle activity in your resting right hand. In the following block, please try to suppress this involuntary activity again as much as possible.

**Table 2:** Statistical table

Data structure	Type of test	Power
Normal distribution	Repeated-measures ANOVA	Frequentist 95% confidence interval
Normal distribution	Pearson correlation	95% confidence interval (estimated using 1000 permutations)

**Table 3:** Results of rmANOVAs (*n* = 20) on log-transformed PSD for each frequency band of interest: δ (1–3 Hz), θ (4–8 Hz), and α (9–12 Hz)

Freq-Band	Factor	df	*F*	*p*	*η²_p_*
δ	ROI	1.45	1.455	0.247	0.071
	Time	1.00	5.864	0.026^*^	0.236
	ROI × Time	2.00	3.706	0.034^*^	0.163
θ	ROI	1.33	0.907	0.379	0.046
	Time	1.00	1.627	0.217	0.079
	ROI × Time	2.00	0.579	0.566	0.030
α	ROI	2.00	31.171	9.720 × 10^−9^*	0.621
	Time	1.00	5.946	0.025^*^	0.238
	ROI × Time	2.00	0.711	0.497	0.036

* significance (*p* < 0.05).

### Data acquisition

**Figure 1.**
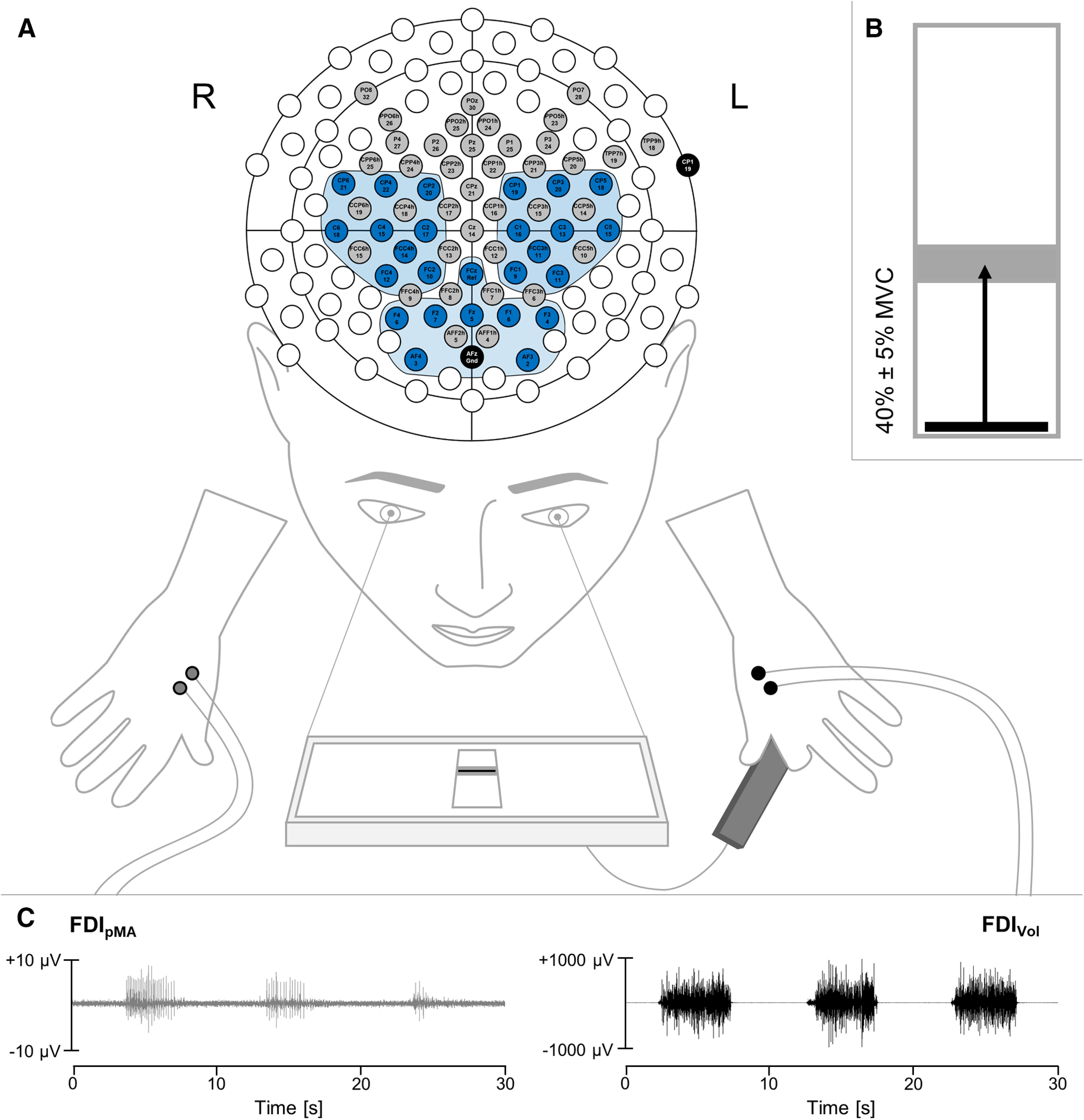
Experimental setup depicting the unilateral motor task combined with EEG and EMG measurements. ***A***, The motor task consisted of unilateral isometric contractions of the left hand through a custom-build force sensor while the right hand was resting in the prone position. For EEG measurements, 64 electrodes were mounted individually on a 128-channel-electrode-cap to densely cover frontal and bilateral sensorimotor cortices (blue and gray electrodes). Predefined ROIs (blue electrodes): left ROI (FCC3h, FC1, FC3, C1, C3, C5, CP1, CP3, CP5), right ROI (FCC4h, FC2, FC4, C2, C4, C6, CP2, CP4, CP6), and frontal ROI (AF3, AF4, F1, F2, F3, F4, Fz, FCz). ***B***, Participants had to vertically move a cursor on a computer screen repetitively (in total 150 times) into a target field by applying pinch force to the sensor (set to require 40 ± 5% of MVC force). The target field was visible for 5 s and was followed by a 5-s rest period during which muscles should be completely relaxed. The experiment was divided into five blocks of 30 contractions each. ***C***, EMG was recorded from the bilateral FDI muscle of the voluntary contracting hand (FDI_Vol_) and the mirroring hand (FDI_pMA_). Please note the different scaling of the EMG traces demonstrating the subliminal nature of pMA. *Figure Contributions:* D. Maudrich designed the figure.

EMG data were recorded using a BrainAmp ExG amplifier system (Brain Products GmbH). Skin preparation consisted of shaving, slight abrasion of superficial keratin layers, and cleaning with alcohol. Sintered Ag/AgCl electrodes (4 mm in diameter) were used in a monopolar setup mounted on bilateral first dorsal interossei muscles of the actively contracting hand (FDI_Vol_) and mirror hand (FDI_pMA_). Reference electrodes were placed on the right and left processus styloideus radii, respectively. One ground electrode was placed on the right epicondylus lateralis humeri. This bilateral setup allowed us to capture voluntary muscle activity of the actively contracting muscles as well as involuntary occurring pMA of the homologous resting muscles in a time-locked manner. EMG data were recorded with a sampling frequency of 1000 Hz. EEG data were acquired using a wireless 64-channel EEG system (LiveAmp, Brain Products GmbH) with an active electrode setup (actiCAP, Brain Products GmbH). Electrodes were mounted individually on an electrode-cap to densely cover bilateral sensorimotor cortices (Fig. 1*A*). Ground and reference electrodes were placed on AFz and left processus mastoideus, respectively. Electrode impedance was kept below 5 kΩ throughout the experiment. Data were continuously recorded with a sampling frequency of 500 Hz. EMG and EEG signals were segmented around the trigger at the start of every contraction performed by the participant, resulting in a total of 150 epochs each lasting 5 s.

### Data processing

All data analyses were performed in MATLAB (v. R2019b, The MathWorks Inc.) using built-in functions, the BBCI toolbox (Blankertz et al., 2016), and the EEGLAB toolbox (Delorme and Makeig, 2004).

EMG signals were first decimated to 500 Hz after low-pass filtering [Chebyshev type I (IIR) eighth-order filter, cutoff 200 Hz]. Furthermore, EMG data were high-pass (fourth-order Butterworth, cutoff 20 Hz) and band-stop filtered at 50 Hz (eighth-order Butterworth) to remove power-line noise. Both EMG signals (FDI_Vol_ and FDI_pMA_) were full-wave rectified, overlaid, and time locked to preserve the temporal relationship between voluntary and involuntary muscular activity. Mean EMG amplitudes of FDI_Vol_, as well as FDI_pMA_, were computed by the estimation of root mean square (RMS) values (window length, 50 ms). Mean EMG amplitudes of the FDI_Vol_ were computed over the time window defined by the externally triggered start and end of each contraction. Latency of pMA was subsequently defined automatically during the delay between burst-onset of FDI_Vol_ and the time point at which muscular activity in the contralateral (resting) FDI_pMA_ exceeded, for a time window of at least 10 ms, a threshold of its mean baseline activity (1000 ms pre-FDI_Vol_ burst onset) + 2 SD (Maudrich et al., 2019). Concerning the amplitude of pMA, latencies were taken into account so that mean EMG amplitudes of FDI_pMA_ were computed over the time window from the determined point of significant elevation of neuromuscular activity until the muscle offset of FDI_Vol_. Mean EMG amplitudes were additionally estimated for pre contraction epochs (4-s rest periods between consecutive contractions) for FDI_Vol_, as well as FDI_pMA_. The absence of overt muscle activity in these epochs was checked and confirmed visually, i.e., the relaxation of participants between each consecutive contraction. This control condition was implemented to test for systematic fatigue-related or attention-related changes in baseline EMG amplitudes across the experiment. All EMG amplitudes were normalized with respect to individual MVC values measured at the beginning of every session for each hand separately. MVC values were estimated by the mean EMG activity over a time window of 500 ms during maximum unilateral force production for FDI_Vol_ and FDI_pMA_, respectively. For each participant mean amplitudes of FDI_Vol_ and FDI_pMA_ were averaged block wise across all 30 contractions (30 precontraction epochs) before statistical analysis. The same procedure was performed for latencies of FDI_pMA_.

EEG data were first bandpass filtered (fourth-order Butterworth, 1–100 Hz). Subsequently, independent component analysis (infomax algorithm; Bell and Sejnowski, 1995) implemented in EEGLAB was used to remove artifact components related to eye movement, eye blinking, and muscle activity (Jung et al., 2000). On average, 5.2 ± 2.5 components were removed per participant. Following Laplacian spatial filtering, power spectral densities (PSDs) were calculated for 30 concatenated epochs of each block (corresponding to 30 voluntary muscle contractions), for each of the 64 EEG channels separately, employing Welch’s method with a Hamming window of 500 samples and an overlap of 50%. Resulting PSDs were subsequently normalized to individual total power (1–100 Hz). Next, relative power within distinct frequency bands of interest (δ: 1–3 Hz, θ: 4–8 Hz, α: 9–12 Hz) was calculated by summation of respective frequency bins and extracted for each participant and electrode separately. Frequency bands of interest were chosen based on previous studies investigating motor inhibitory processes (Kaiser et al., 2019) and executive processes involved in lateralized inhibition of symmetrical contractions (Tisseyre et al., 2019). Subsequently, PSDs were averaged across three regions of interest (ROIs; Fig. 1*A*): left sensorimotor ROI (FCC3h, FC1, FC3, C1, C3, C5, CP1, CP3, CP5), right sensorimotor ROI (FCC4h, FC2, FC4, C2, C4, C6, CP2, CP4, CP6), and frontal ROI (AF3, AF4, F1, F2, F3, F4, Fz, FCz). Finally, relative power within each frequency band (δ, θ, α), block (BL–IV-block), and ROI (FRONTAL, LEFT, RIGHT) was log-transformed before statistical analyses.

The procedure was repeated for precontraction epochs (5-s rest periods between consecutive contractions) for all blocks. This control condition was implemented to test whether changes in spectral power between naive and informed blocks represent general longitudinal effects or are indeed specific to voluntary inhibitory mechanisms during the task execution.

### Statistical analysis

Normality of all parameters (i.e., RCT, amplitude and latency of EMGs, log-transformed power) was assessed and confirmed through Lilliefors testing (α = 0.05). All further statistical analyses were performed using JASP (version 0.13.1; JASP Team, 2020). Block-wise differences in RCT were investigated using a repeated-measures ANOVA with the within-subject factor BLOCK (BL, I, II, III, IV). Bonferroni adjusted *p* values of *post hoc* comparisons are reported within the results. For FDI_Vol_ and FDI_pMA_ (contraction and precontraction epochs), block-wise changes in amplitude were investigated using separate repeated-measures ANOVAs with the within-subject factor BLOCK (BL, I, II, III, IV). Here, the significance level was Bonferroni adjusted for four pairwise *post hoc* comparisons (BL vs I, BL vs II, BL vs III, BL vs IV; *p*_adj_ < 0.0125). The same model was implemented to test for block-wise changes in latency of FDI_pMA_. In the case of a violation of the sphericity assumption, Greenhouse–Geisser correction was implemented.

In order to compare PSD during unilateral contractions performed in a naive versus informed state, two conditions were defined for each frequency band and ROI separately: (1) power of BL-block (NAIVE) and (2) power of I-block (INHIBIT). The comparison of these two conditions reflects immediate power changes accompanying initial behavioral inhibition. Changes in log-transformed spectral power (for contraction epochs as well as precontraction epochs separately) were subsequently analyzed through two-way repeated-measures ANOVAs with the within-subject factors ROI (FRONTAL, LEFT, RIGHT) and CONDITION (NAIVE, INHIBIT) for each frequency band separately (δ, θ, α). In the case that sphericity was violated, Greenhouse–Geisser correction was implemented. Simple main effect and pairwise *post hoc* analyses were conducted in case of significant interaction effects.

To test our hypothesis that the amplitude of pMA observed during naive (BL) and informed blocks (I, II, III, IV) is inversely associated with the individual δ power in the frontal ROI for each respective block, linear regression analyses were conducted on all data per block. δ Power served as dependent variables and corresponding pMA amplitudes served as predictors. Next, we assessed normality of residuals by way of Lilliefors testing (α = 0.05). All residuals were normally distributed. Thereafter, Pearson correlation coefficients were estimated for each block. To evaluate the significance of our results, δ power and pMA amplitude vectors were randomly permuted, resulting in a distribution of Pearson correlation coefficients from 1000 permutations (Manly, 2006). The Pearson correlation coefficients were deemed significant when they eclipsed the 95th percentile of permuted data. Family-wise error corrected *p* values of Pearson correlation coefficients are reported with the results. Additionally, Fisher’s *r* to *z*-transformation was implemented to test for a significant difference in the correlation coefficients of I-block and IV-block (Fisher, 1921).

The same procedure was used to estimate the association between participants’ initial pMA amplitudes (BL-block) and immediate rates of inhibition [pMA amplitude BL-block–I-block (%)].

For all statistical comparisons, *p* < 0.05 was considered significant. All *p* values Bonferroni adjusted for multiple comparisons are reported with the results.

An overview of statistical methods is provided in Table 2.

## Results

### Behavioral parameters

All participants consistently achieved the performance goal of the motor task by reaching the target field (corresponding to 40 ± 5% MVC) in every 5-s contraction during the experiment (i.e., 150 contractions). Repeated-measures ANOVAs indicated a significant effect for the factor BLOCK on RCT (*F*_(2.1,39.9)_ = 11.975, *p* = 6.660 × 10^−5^, η_p_^2^ = 0.387). *Post hoc* comparison showed that RCT significantly increased from BL to II (MD = 257.098, SE = 48.409, *p* = 1.049 × 10^−5^, *d* = 1.188) and stayed elevated thereafter compared with BL during III (MD = 262.977, SE = 48.409, *p* = 6.442 × 10^−6^, *d* = 1.215) and IV (MD = 274.906, SE = 48.409, *p* = 1.049 × 10^−5^, *d* = 1.270).

### EMG parameters

**Figure 2.**
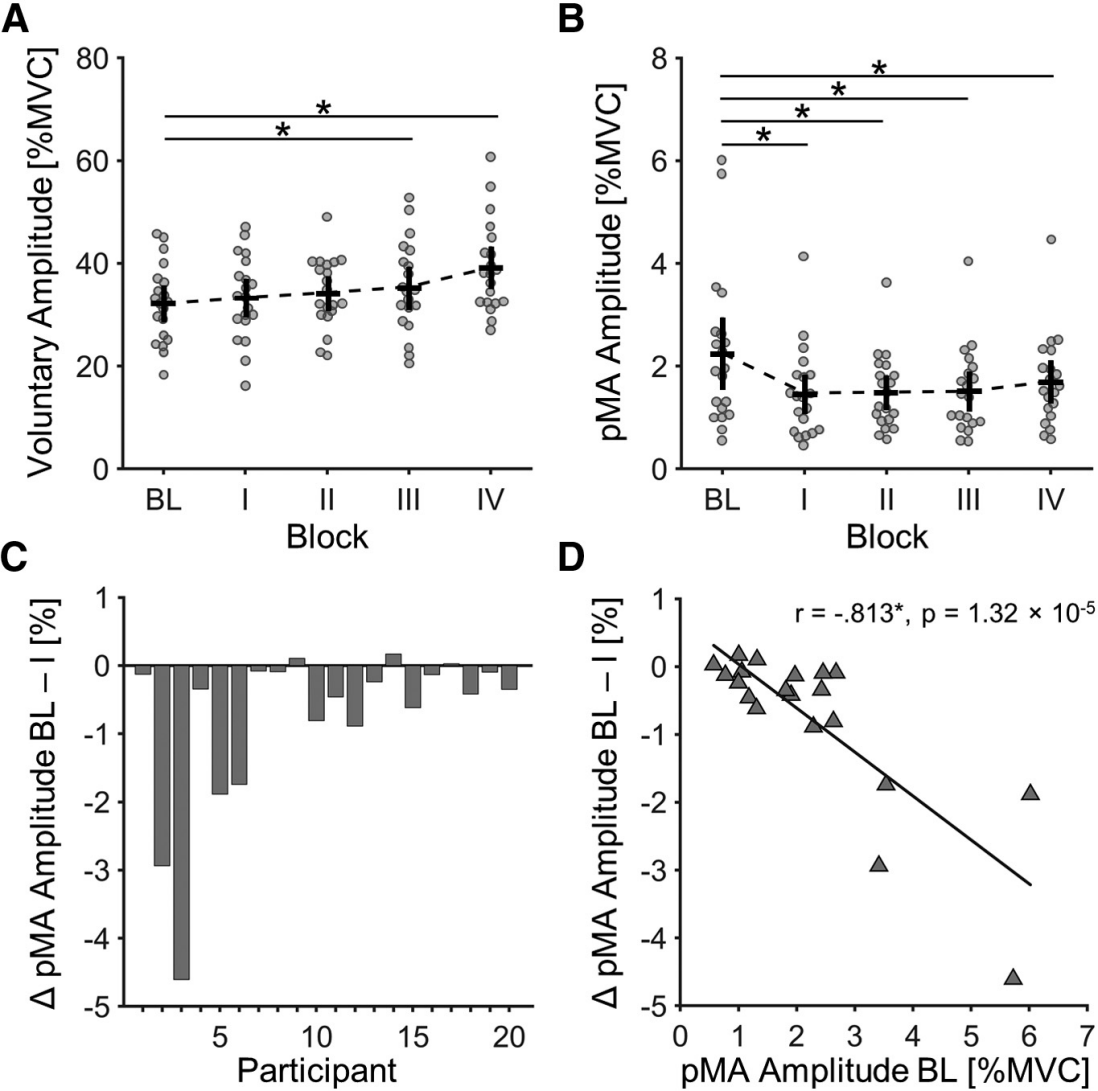
EMG results. ***A***, Block-wise EMG amplitude of voluntary muscle contractions (FDI_Vol_). Bars around the mean indicate 95% confidence interval of the mean. Asterisks indicate significant *post hoc* pairwise comparisons, significance level Bonferroni adjusted to *p* < 0.0125. ***B***, Block-wise EMG amplitude of pMA (FDI_pMA_). Bars around the mean indicate 95% confidence interval of the mean. Asterisks indicate significant *post hoc* pairwise comparisons, significance level Bonferroni adjusted to *p* < 0.0125. ***C***, Individual percentage change in amplitude of FDI_pMA_ from BL-block to I-block for each participant. ***D***, Significant inverse relationship between initial levels of pMA (pMA amplitude in BL-block) and the percentage change in pMA amplitude from BL-block to I-block. Asterisks indicate significant correlation after permutation testing (1000 permutations). BL, baseline block; I, first inhibition block; II, second inhibition block; III, third inhibition block; IV, fourth inhibition block.

Repeated-measures ANOVAs indicated a significant effect for the factor BLOCK on mean amplitudes of FDI_Vol_ (*F*_(2.517,47.832)_ = 10.829, *p* = 4.092 × 10^−5^, η_p_^2^ = 0.363) and FDI_pMA_ (*F*_(1.460,27.740)_ = 7.140, *p* = 0.006, η_p_^2^ = 0.273). More specifically, *post hoc* analyses showed a significant increase in amplitude from BL to III (MD = 0.032, SE = 0.012, *p* = 0.006, *d* = 0.630) and BL to IV (MD = 0.070, SE = 0.012, *p* = 4.748 × 10^−8^, *d* = 0.966) for FDI_Vol_ (Fig. 2*A*). In all blocks where participants were informed about the nature of the experiment, the amplitude of FDI_pMA_ was significantly reduced, compared with the naive BL (Fig. 2*B*): BL to I (MD = −0.008, SE = 0.002, *p* = 2.851 × 10^−5^, d = –0.652), BL to II (MD = −0.008, SE = 0.002, *p* = 4.058 × 10^−5^, d = –0.645), BL to III (MD = −0.07, SE = 0.002, *p* = 5.683 × 10^−5^, d = –0.677), and BL to IV (MD = −0.006, SE = 0.002, *p* = 0.006, d = –0.515). Mean EMG amplitudes of FDI_Vol_ during precontraction epochs showed no difference between blocks, suggesting the absence of systematic changes in EMG baseline over the course of the experiment (*F*_(2.954,56.120)_ = 1.615, *p* = 0.179, η_p_^2^ = 0.078). In case of FDI_pMA_ precontraction epochs, a significant effect of BLOCK was found (*F*_(2.286,43.440)_ = 5.327, *p* = 0.006, η_p_^2^ = 0.219). Corresponding to contraction epochs of FDI_pMA_, mean EMG amplitudes were significantly reduced in all blocks where participants were informed about the nature of the experiment (except for IV-block) compared with BL: BL to I (MD = −0.003, SE = 8.368 × 10^−6^, *p* = 0.006, d = –0.802), BL to II (MD = −0.003, SE = 8.368 × 10^−6^, *p* = 0.002, d = –0.865) and BL to III (MD = −0.003, SE = 8.368 × 10^−6^, *p* = 0.003, d = –0.855). For latency of FDI_pMA_ no significant effect for the factor BLOCK was found (*F*_(4.000,76.000)_ = 1.456, *p* = 0.224, η_p_^2^ = 0.071).

### δ Band

**Figure 3.**
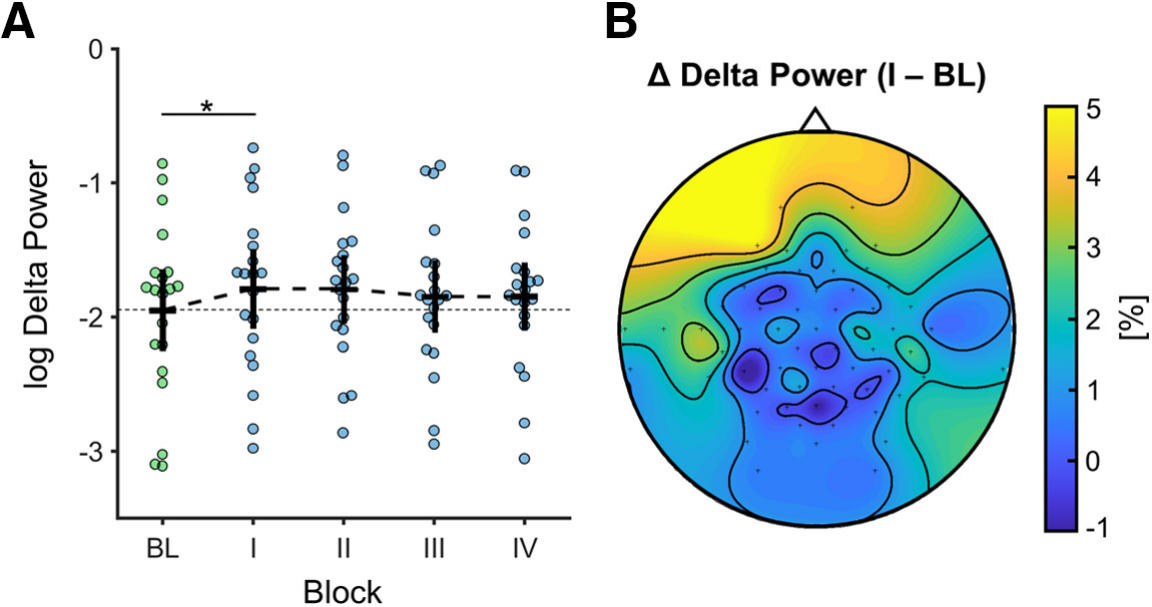
Spectral power change in the δ band. ***A***, Significant increase in log-transformed δ power in frontal ROI from BL-block to I-block, reflecting immediate power changes accompanying initial behavioral inhibition. Bars around the mean indicate 95% confidence interval of the mean. Asterisks indicate significant pairwise comparison. ***B***, Topographical map showing grand averaged percentage increase in δ power in frontal electrodes (contrast: I-block – BL-block). BL, baseline block; I, first inhibition block; II, second inhibition block; III, third inhibition block; IV, fourth inhibition block.

With regards to spectral power in the δ band (1–3 Hz) during contraction epochs, a repeated-measures ANOVA revealed a significant interaction effect for the factors ROI × CONDITION (*F*_(2,38)_ = 3.706, *p* = 0.034, η_p_^2^ = 0.163, Table 3). A simple main effect analysis (simple effect factor: CONDITION; moderator factor: ROI) suggests the interaction was present in the FRONTAL ROI (*p* = 0.003). Pairwise *post hoc* comparisons confirmed a significant increase in δ power from the NAIVE to INHIBIT condition in the FRONTAL ROI (MD = 0.159, SE = 0.046, *p*_bonf_ = 0.020, *d* = 0.777; Fig. 3). Furthermore, a significant main effect of the factor CONDITION (*F*_(1,19)_ = 5.864, *p* = 0.026, η_p_^2^ = 0.236) was found, with spectral power being significantly higher during INHIBIT compared with NAIVE (MD = 0.092, SE = 0.038, *p*_bonf_ = 0.026, *d* = 0.541). No significant main or interaction effects were found for precontraction epochs (all *p* > 0.05).

### θ Band

In the θ band (4–8 Hz) no significant main or interaction effects were found, neither for contraction nor precontraction epochs (all *p* > 0.05).

### α Band

For the α band (9–12 Hz) in contraction epochs, significant main effects for ROI (*F*_(2,38)_ = 31.171, *p* = 9.720 × 10^−9^, η_p_^2^ = 0.621) and CONDITION (*F*_(1,19)_ = 5.946, *p* = 0.025, η_p_^2^ = 0.238) were found. Pairwise *post hoc* analyses indicated significantly lower power in the FRONTAL ROI compared with the LEFT (MD = −0.387, SE = 0.065, *p*_bonf_ = 2.887 × 10^−5^, d = −1.334) and RIGHT ROIs (MD = −0.367, SE = 0.058, p_bonf_ = 1.475 × 10^−5^, d = −1.406). Furthermore, a significant increase in power from the NAIVE to INHIBIT condition was revealed (MD = 0.080, SE = 0.033, *p*_bonf_ = 0.025, *d* = 0.545). However, no significant interaction effect was found.

For precontraction epochs, a similar result was obtained. Significant main effects for ROI (*F*_(1.458,27.698)_ = 48.306, *p* = 1.042 × 10^−8^, η_p_^2^ = 0.718) and CONDITION (*F*_(1,19)_ = 10.551, *p* = 0.004, η_p_^2^ = 0.357) were found. Pairwise *post hoc* analyses indicated significantly lower power in the FRONTAL ROI compared with the LEFT (MD = −0.447, SE = 0.063, *p*_bonf_ = 2.782 × 10^−6^, d = −1.589) and RIGHT ROIs (MD = −0.424, SE = 0.052, *p*_bonf_ = 3.937 × 10^−7^, d = −1.818). Furthermore, a significant increase in power from the NAIVE to INHIBIT condition was revealed (MD = 0.091, SE = 0.028, *p*_bonf_ = 0.004, *d* = 0.726). Again, no significant interaction effect was found.

### Correlation analysis

**Figure 4.**
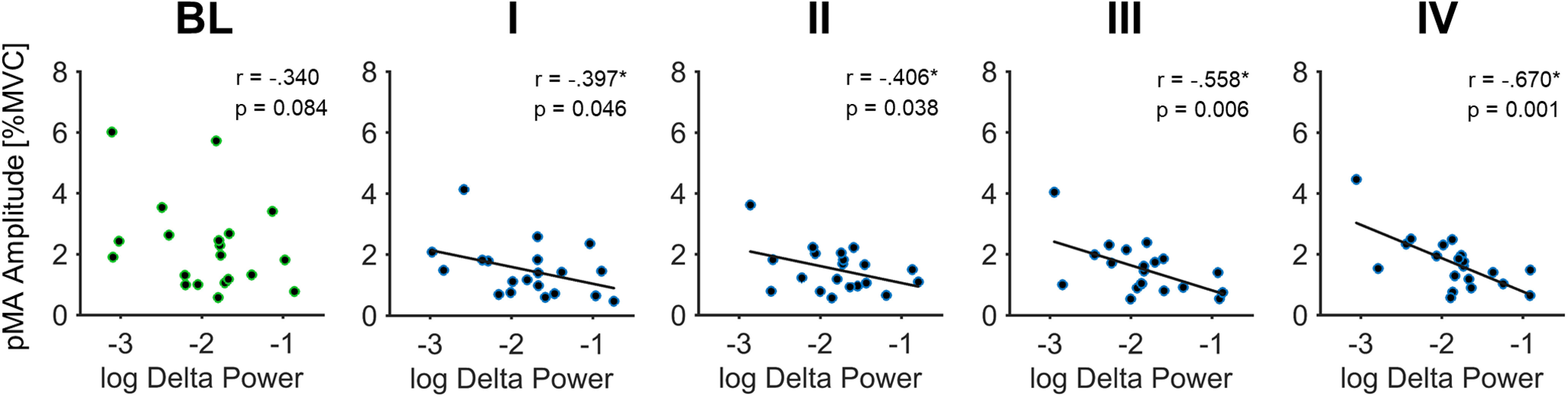
Inverse relationship between frontal δ power and the amplitude of pMA. Bivariate Pearson correlation depicted for each contraction block separately. The inverse association reached significance once participants were instructed to inhibit pMA (I-block). Asterisks indicate significant correlation after permutation testing (1000 permutations). BL, baseline block; I, first inhibition block; II, second inhibition block; III, third inhibition block; IV, fourth inhibition block.

During the naive BL, frontal δ power and the amplitude of pMA were not significantly associated (*r*_(20)_ = –0.340, *p* = 0.084). Once participants started to inhibit pMA, a significant negative correlation emerged during I (*r*_(20)_ = –0.397, *p* = 0.046), II (*r*_(20)_ = –0.406, *p* = 0.038), III (*r*_(20)_ = –0.558, *p* = 0.006) and IV (*r*_(20)_ = –0.670, *p* = 0.001; Fig. 4). Pearson correlation coefficient of I-block and IV-block were not significant different from each other (*z* score = 1.139, *p* = 0.127).

Furthermore, we found a significant negative correlation between initial levels of pMA (pMA amplitude in I-block) and the percentage change in pMA amplitude from BL-block to I-block (*r*_(20)_ = –0.813, *p* = 1.32 × 10^−5^). That means that participants with higher initial pMA amplitudes showed the highest rates of inhibition from BL-block to I-block.

## Discussion

Using a multimodal approach by combining EMG and EEG recordings, we provide novel evidence of pMA suppression in healthy adults. Suppression was accompanied by increased δ power in frontal areas. We show that the amplitude of pMA can be voluntarily inhibited during unilateral isometric contractions of intrinsic hand muscles. This was accomplished by asking participants to do so, after informing them about the presence of pMA and offering a basic understanding of pMA mechanisms through a standardized protocol. Importantly, significant inhibition of pMA was observed despite the absence of any online feedback during task execution and without special training.

A consistent and robust observation is that the amplitude of pMA is modulated by force demands of unilateral movements, i.e., increases with rising force requirements (Todor and Lazarus, 1986; Aranyi and Rosler, 2002; Shinohara et al., 2003; van Duinen et al., 2008; Sehm et al., 2010, 2016; Maudrich et al., 2017). To avoid any influence of this relationship, the force level in this study was kept constant (40 ± 5% MVC). In addition, the quality of task performance, i.e., consistently achieving the desired force level, is expected to have an influence on pMA amplitude. However, all subjects reached the target field in each trial. Together, we were, therefore, able to keep two factors that mainly influence pMA amplitude constant. Interestingly enough, a significant reduction of pMA amplitude was achieved in all informed force blocks. As we intentionally interfered with the force dependency of pMA by directing participants’ attention toward the presence of pMA and asking them to inhibit any involuntary co-activation, this observation might suggest executive control, through directed attention, exerts inhibitory drive on involuntary motor output, suppressing pMA. In support of this, it has been shown in Go/No-Go reaction tasks, that primary motor cortex excitability is suppressed (Hoshiyama et al., 1996, 1997; Leocani et al., 2000), and short intracortical inhibition (SICI) is enhanced (Waldvogel et al., 2000; Sohn et al., 2002) during voluntary inhibition of movements, providing a potential neurophysiological basis for voluntary inhibition of pMA. Interestingly, we observed different rates of inhibition between participants (Fig. 2*C*). In conjunction with this, we found a strong negative correlation between initial levels of pMA (pMA amplitude in I-block) and the percentage change in pMA amplitude from BL-block to I-block (Fig. 2*D*). This suggests that participants with higher initial pMA amplitudes showed the highest rates of inhibition. However, this correlation must be interpreted with caution. In some participants, very low initial pMA values and inhibition rates were present, suggesting on the one hand that pMA cannot demonstrate a strong decrease because of the “floor effect.” On the other hand, it might be possible that the signal-to-noise ratio of the surface EMG recordings could be limiting the potential for inhibition of participants with initially low pMA amplitudes in the present experiment. Intramuscular needle EMG might be better suited to detect the most subtle involuntary discharges of motor units and could, therefore, be used in future studies to resolve the question of whether pMA can be completely eliminated.

Investigations of right-handed adults, using similar isometric contractions to our implemented task, reported no side differences in pMA between right and left-hand contractions (Addamo et al., 2009; Koerte et al., 2010; Sehm et al., 2016; Maudrich et al., 2017, 2018). Therefore, it seems reasonable to assume that the choice of the contracting hand in the current study (left), most likely did not influence the obtained result. However, future studies are needed to replicate the voluntary inhibition of pMA during dominant hand contractions in right-handed adults.

In previous investigations regarding pMA, it has been ensured that participants were naive to the study aim and generally the phenomenon of motor overflow to prevent any voluntary inhibition (Post et al., 2009; Maudrich et al., 2019). In the present experiment it seems remarkable that by shifting attention to the involuntarily contracting hand and establishing a basic understanding of pMA, most of the participants were able to significantly inhibit the amplitude of pMA in successive contraction blocks without any online feedback about their performance or special training. After the experiment, participants were asked to describe their self-chosen strategy used to inhibit pMA. While many different and highly individualized strategies have been mentioned, a main common denominator can be inferred. All participants, in one way or another, mentally focused on non-activation of the non-performing hand during each contraction of the performing hand. Thus, constantly paying attention to muscle relaxation in non-performing limbs might be a prerequisite for successful pMA inhibition besides being aware of its presence. However, no clear relation between individually employed strategies and participants inhibition rates is recognizable so that a decisive conclusion cannot be drawn from this dataset. Another strategy that was not reported but is manifested in increased RCTs during the experiment might be to perform more deliberate, less explosive contractions of the performing hand. Slower contractions of the performing hand, without changing the required target force level, might have allowed for easier suppression of pMA in the non-performing hand.

In line with the behavioral inhibition, we observed an increase in relative δ power from BL-block to the consecutive I-block in the frontal ROI (sensor space). This task-related increase in δ power was most likely not because of general longitudinal or non-stationarity effects during the experiment as our control comparison of non-task-related epochs (directly preceding muscle contractions) failed to reach significance. Previously, it has been hypothesized that there is a connection between δ-oscillations and suppression of unwanted neural activity (Vogel et al., 1968). Furthermore, studies have shown that increases in δ power in frontal regions are associated with behavioral inhibition (Kamarajan et al., 2004; Knyazev, 2007; Putman, 2011), and it has been proposed that midfrontal δ power might serve as a selective marker for motor inhibition (Kaiser et al., 2019). Other investigations incorporating Go/No-Go tasks have observed an increase in δ activity during inhibition of movement in frontal electrodes (Harmony et al., 2009). Moreover, another study investigating the neurocognitive effects of alcoholism proposed that decreased δ and θ power in frontal regions, associated with No-Go processing, might suggest a deficient inhibitory control and information-processing mechanism in alcoholics (Kamarajan et al., 2004). In addition, we found a significant inverse association between the amplitude of pMA and frontal δ power that emerged once participants were instructed to inhibit pMA and persisted throughout all successive force blocks (Fig. 4). Overall, these results suggest that δ power in frontal regions might reflect executive processes relating to inhibitory control (Chambers et al., 2009; Munakata et al., 2011), modulating unintentional motor output, in our case pMA. Importantly, the fact that participants were able to suppress pMA immediately after they were instructed indicates that no special training was required. Thus, a reduction of involuntary motor output had rather a categorical character akin to a termination of a given behavioral output. Such regulation of behavior is a hallmark of executive functions, particularly inhibitory control, which often has a character of a veto, once undesirable consequences of the behavior become known (Kühn et al., 2009). In such a case, the over-riding of pMA is similar to not performing a Go-response in a No-Go paradigm, once the participant is made aware that she/he was committing this response but were not yet aware of such behavior. Bringing the presence of pMA to their attention thus requires them to perform a Go-response with one hand (voluntary contraction) while simultaneously preventing this response with the other hand (involuntary muscle activation).

Contrary to our hypothesis, we did not observe changes in θ power from BL-block to I-block. This seems surprising, as a previous investigation found a θ power increase associated with lateralized inhibition of symmetrical movements in the fronto-mesial area, attributed to executive processes (Tisseyre et al., 2019). This contradiction may be explained by differences between our task and those from previous studies showing θ power increases during motor inhibition. Such increases in θ power were often demonstrated during voluntary response inhibition paradigms (Cohen et al., 2011; Andreou et al., 2017). In this context, voluntary response inhibition is closely related to feedback on task performance, meaning that inhibitory strategies and their success rates can be assessed and optimized during task performance (Yamanaka and Yamamoto, 2010). Thus, this ability to strategically fine-tune inhibitory strategies (Kirmizi-Alsan et al., 2006; Yamanaka and Yamamoto, 2010; Tisseyre et al., 2019) is a major difference compared with the task we employed in this study. Because of the inherent characteristics of the motor task in the present study, where participants were entirely unaware regarding successful inhibition of pMA throughout the experiment, strategic fine-tuning is unlikely to have occurred because of participants’ uncertainty regarding successful inhibition. This mechanistic difference may serve as an explanation for the absence of an increase in θ power as participants could not have been certain regarding successful pMA inhibition and, therefore, stable θ dynamics, reflecting voluntary inhibitory response mechanisms did not emerge.

With regards to α-oscillations, which among other functions generally reflect inhibitory control mechanisms in the motor domain (Klimesch et al., 2007; Sauseng et al., 2013), we observed overall global power increases from naive to informed contractions regardless of ROI. Furthermore, this global increase in α power was also observed in our control analysis of non-task related epochs. An increase in α power over time has previously been associated with the level of sustained attention and fatigue (Boksem et al., 2005; Craig et al., 2012). Recently, multiple endogenous non-stationarity processes in α band activity, i.e., an increase in power, have been shown to occur over time (Benwell et al., 2019). Therefore, the global α power increase observed in the present study most likely reflects the longitudinal effects of fatigue, sustained attention, or endogenous non-stationarity processes. However, the absence of specific changes in α power further supports our interpretation that inhibition of pMA in this study is mediated by executive control, rather than by purely motor inhibition processes.

As a limitation of the present study, we are not able to disentangle the distinct effects of directed focus on pMA presence and actual inhibitory processes on the observed behavioral suppression of pMA. One previous study observed that pMA remained unchanged after young adults were informed of it but asked to ignore their motor overflow. Only when participants were requested to inhibit involuntary co-activation with and without visual feedback were they able to reduce pMA (Addamo et al., 2010). While this within-subject study protocol seems appropriate to disentangle the effects of directed focus and actual inhibitory processes on pMA suppression in general, we suggest that a between-subject protocol might be an alternative. As part of this between-subject protocol, one intervention group would be informed about pMA presence but instructed to ignore it, while a separate intervention group is additionally instructed to actively inhibit pMA. A control group would be naive regarding pMA throughout the experiment. This experimental design could be repeated over several days to investigate the stability of these mechanisms or even learning effects in pMA inhibition over time. However, as stated before, force measurements are insufficient in capturing neurophysiological aspects of pMA in its entirety, rendering future studies incorporating EMG measurements necessary to answer these questions raised above.

In conclusion, we provide novel evidence for voluntary suppression of pMA in healthy adults. As proposed by others, the knowledge of voluntary pMA modulation via attentional processes might be helpful for therapeutic applications in the context of neurorehabilitation, i.e., for the development of cognitive-behavioral strategies for the treatment of mirror movements (MM) that are not a result of major anatomic abnormalities (e.g., uncrossed corticospinal tract; Addamo et al., 2007, 2009). One possible intervention might be to implement online feedback of involuntary muscular activity to voluntarily decrease the level of EMG and even possibly the occurrence of overt MM, as it has been demonstrated previously in brain-injured young adults (Lazarus, 1992). However, the transfer of pMA-mechanisms to pathologic MM remains elusive and requires further research. Nevertheless, this inhibitory feedback-based approach appears additionally promising in the context of counteracting the age-related increases in pMA, which might help prevent the decline in motor performance documented in the elderly.

## Synthesis

Reviewing Editor: Macià Buades-Rotger, University of Luebeck Decisions are customarily a result of the Reviewing Editor and the peer reviewers coming together and discussing their recommendations until a consensus is reached. When revisions are invited, a fact-based synthesis statement explaining their decision and outlining what is needed to prepare a revision will be listed below. The following reviewer(s) agreed to reveal their identity: Elisa Kallioniemi, Steven Jerjian.

The authors used EMG and EEG measurements to provide new insights into the capacity for inhibitory control over physiological mirror activity (pMA), the involuntary activation of (homologous) muscles in the non-performing hand during a unimanual movement. The paper investigates whether healthy young humans can attenuate pMA following explicit instruction, as measured by EMG activity during a simple force task. The paper also addresses whether attenuation is correlated with changes in spectral power in low-frequency bands within frontal and sensorimotor ROIs. The results show a sustained decrease in pMA after baseline, and an accompanying increase in frontal ROI delta power, a frequency band associated with inhibitory control.

The paper to be interesting, clearly written, and provides novel insights into the physiological mechanisms of pMA, and the capacity for its overt suppression in healthy subjects. I will thus recommend it for publication provided that the authors address the issues raised by reviewers, which are detailed in the following.

Major

- Lines 55-57: is pMA a “contributing factor” or an epiphenomenon/marker/consequence of age-related declines in motor performance? Are there studies suggesting a causal direction? This is a crucial point: if pMA were just a consequence of motor decline, sentences assuming assuming it’s the therapeutic value of voluntary pMA suppression should be deleted, such as in the significance statement (lines 27-30): “a possible strategy to counteract the age-related increase in mirror activity with beneficial effects on motor performance during healthy aging.”

- When comparing EMG and EEG, did the authors take into account the approximate 25ms delay it takes neural activity to travel from the motor cortex to the peripheral muscle?

- The authors do not mention about baseline correction for the EEG data. Was the data corrected before calculating spectral power?

- The authors should provide the script for the explanation and verbal instructions to suppress pMA.

- All subjects were right-handed, though they used the left-hand as the performing hand (FDIvol), with the dominant FDI probed for pMA. This is in contrast to one of the key references (Maudrich et al. 2019), in which right-handed subjects performed a similar task with their dominant right hand. I initially wondered whether there may be some asymmetry in pMA in humans in relation to hand dominance, although a brief look at the literature suggests this is not the case. Nonetheless, the authors should explain the rationale for the choice of performing hand, and address whether this is relevant in the Discussion.

- The text suggests that the onset of the target presentation in each 5s ‘trial’ served as the go cue for subjects to make their movement. Was there any measure of reaction time on each trial? It is not mentioned whether the subjects were debriefed on their self-chosen mental strategy, but one possible strategy for suppressing pMA might be to consciously make slower, more deliberate movements, which would manifest in longer RTs in the performing hand. Was this the case?

- On a related note, it would be interesting to discuss what the authors consider to be potential strategies for inhibiting pMA (see also point 2), and the implications of this for how pMA might be reduced in pathological or higher amplitude pMA in healthy people. Given subjects were free to choose their own strategy, it seems likely that the effectiveness of different strategies could account for some of the high inter-subject variability shown in Fig. 2b.

- Line 110: “during which muscles should be completely relaxed”. The authors perform some analysis of non-task related epochs for the EEG data, but did they confirm from the actual data that subject EMG (in both hands) was consistently absent during these periods as well, and that there were no systematic (e.g. fatigue/attention-related) changes over the course of the experiment?

- The stated goal of the task is to minimize pMA in the non-performing hand while maintaining consistent FDIvol EMG (L118-119). This seems to be a critical point - any concomitant reductions in FDIvol EMG would suggest subjects can inhibit pMA simply by reducing overall motor drive, rather than engaging any specific inhibition of the non-performing hand. In actual fact, the authors report increases in FDIvol during III and IV, in contrast to the decreases in pMA. This is suggested in the discussion to be due to fatigue effects as shown previously but is somewhat conflicting with the goal stated in the introduction. Overall, the details and presented data on FDIvol data were lacking. It would be important to know that subjects were indeed consistently achieving the desired force level with the performing hand in order to rule out changes here as a confound. Was there any measure of subject performance in the vertical cursor target task itself? And were there any within-subject relationships in changes in FDIvol and FDIpma amplitudes over the course of the experiment?

- The authors suggest that subjects with minimal changes in pMA over the course of the task may have been due to floor effects, since pMA is already a fraction of MVC amplitudes at BL (L335-336). It cannot be inferred from Fig. 2 whether this is indeed the case - were the subjects in 2b with minimal changes also the ones with low BL pMA amplitude (in 2a)? It should be fairly straightforward to check this within the data in order to support the authors’ suggestion.

- In the correlation analysis (Fig. 4), although the BL block is not significant and the I block is, this does not strictly support the conclusion that the inverse correlation emerges in the I block, given how similar the Pearson correlation values are. Block III and IV do show progressively stronger correlations, although here, the effect of the outlier at -3, +4 (presumably the same subject in all panels) is concerning. A more convincing demonstration of a genuinely strengthening correlation across blocks would require direct comparisons between blocks (e.g. Steiger’s Z test), as well as an estimation method robust to outliers (e.g. bootstrapping).

- The authors suggest that the increasingly negative correlation between pMA and log delta power, particularly in III and IV blocks, is due to fatigue and “increased inhibitory demands” (L363-367). The slight increases in pMA between blocks I and IV in Fig. 2 might support this indirectly, but doesn’t this interpretation suggest that larger increases in delta power should also occur in order to maintain pMA suppression as the task progresses? On the other hand, the results presented in Figs. 2 and 3 suggest that the largest delta power increase, and to some extent, the strongest pMA inhibition, both occur between the BL and I block. It is difficult to reconcile these points. At the least, it would help for the authors to present comparisons between BL and the other ‘informed’ blocks as well in Fig. 3. Also, have the authors considered a correlation analysis using the differences between blocks, rather than the individual block values themselves? This may provide better support for a stricter “within-subject” interpretation regarding increasing fatigue levels.

- Line 383: The authors speculate that the reason they did not find effects in the theta band may have been due to task differences from previous studies related to the duration/frequency of contraction. Could the authors expand on why they think these task differences might influence theta power?

- Line 402: The authors note a limitation of their study in dissociating the effects on pMA suppression of directed attention vs inhibitory processes. This is an inevitable consequence of the task design where subjects are told about pMA and encouraged to inhibit it, and there is no control group. While I agree with the authors that EMG measurements are a useful technical advance, it is not clear what kind of control or alternative tests they have in mind here to dissociate the two processes they refer to, other than perhaps a similar protocol to the cited Addamo et al. 2010 paper.

Minor

- Please spell out all acronyms again in the Discussion in order not to tax the reader’s working memory or cause them to move back and forth.

- Line 36: there is no need to introduce the acronym MM here, as it is not used again in the Introduction.

- Figure 1 seems to serve primarily as a methods figure for the EEG electrode and ROI configuration, but also includes EEG results, which are shown in a different form in Figure 3. The rationale for splitting it this way is not clear. As a reader, an alternative figure 1 depicting the task design (perhaps showing some FDIvol/FDIpma activity) would have been helpful.

- Line 121: “Online feedback was not provided.” Does this mean that subjects were not able to see a display showing EMG in their FDI muscles, but were able to see both hands at all times?

- Lines 147, 201, 220, 234...: “analyses“

- Lines 202-210: There seems to be some unnecessary repetition here. Excepting the GG correction, the analyses for the two FDIs appear identical.

- The authors have used the abbreviations BL and I in the figures. It would be clearer to add definitions as well.

- In figure 3B, could the authors add units to the color bar?

## Author Response

Point-by-point reply to the reviews

Manuscript Instructions

1) Statistical Table should be included as a proper Table with appropriate labeling (Table 1, Table 2, etc.)

Our response: We included the Statistical Table with appropriate labeling in the manuscript (i.e. “Table 2”).

2) The species studied is not mentioned in the abstract. Please make sure to update both the abstract in the article file and on the submission form.

Our response: We updated the abstract by adding the species and sex studied. The abstract now reads as follows:

„Physiological mirror activity (pMA), observed in healthy human adults, describes the involuntary co-activation of contralateral homologous muscles during unilateral limb movements. Here we provide novel evidence, using neuromuscular measurements (electromyography), that the amplitude of pMA can be voluntarily inhibited during unilateral isometric contractions of intrinsic hand muscles after informing human participants (10 male, 10 female) about its presence and establishing a basic understanding of pMA mechanisms through a standardized protocol. (...)” [l. 3-9]

---------------------------------------------

Synthesis of Reviews:

Computational Neuroscience Model Code Accessibility Comments for Author (Required): N/A

Significance Statement Comments for Author (Required): N/A

Comments on the Visual Abstract for Author (Required): N/A

The authors used EMG and EEG measurements to provide new insights into the capacity for inhibitory control over physiological mirror activity (pMA), the involuntary activation of (homologous) muscles in the non-performing hand during a unimanual movement. The paper investigates whether healthy young humans can attenuate pMA following explicit instruction, as measured by EMG activity during a simple force task. The paper also addresses whether attenuation is correlated with changes in spectral power in low-frequency bands within frontal and sensorimotor ROIs. The results show a sustained decrease in pMA after baseline, and an accompanying increase in frontal ROI delta power, a frequency band associated with inhibitory control.

The paper to be interesting, clearly written, and provides novel insights into the physiological mechanisms of pMA, and the capacity for its overt suppression in healthy subjects. I will thus recommend it for publication provided that the authors address the issues raised by reviewers, which are detailed in the following.

Major

3) Lines 55-57: is pMA a “contributing factor” or an epiphenomenon/marker/consequence of age-related declines in motor performance? Are there studies suggesting a causal direction? This is a crucial point: if pMA were just a consequence of motor decline, sentences assuming it’s the therapeutic value of voluntary pMA suppression should be deleted, such as in the significance statement (lines 27-30): “a possible strategy to counteract the age-related increase in mirror activity with beneficial effects on motor performance during healthy aging.”

Our response: Thank you for this comment. Observational studies reported higher pMA in older compared to younger adults (Shinohara et al., 2003; Koerte et al., 2010), possibly due to an age-related loss of central inhibition (Carr, 2010). Based on this observation it was speculated that this increase in pMA might be one of many altered patterns of muscle activation in the elderly, negatively impacting manual dexterity and motor performance (Shinohara et al., 2003). However, to our knowledge, an investigation suggesting a causal direction between levels of pMA and motor performance in the elderly is still missing. We agree that suggesting a therapeutic benefit of voluntary pMA inhibition regarding the decline in motor performance of the elderly is a rather strong statement and therefore deleted this part from the manuscript. Nevertheless, we still believe that an inhibitory feedback-based approach might help counteract the age-related increase in pMA in general. The respective parts of the Abstract, Significance statement and Introduction now read as follows:

“Our results provide an initial reference point for the development of therapeutic applications related to the neurorehabilitation of involuntary movements which could be realized through the suppression of pMA observed in the elderly before it would fully manifest in undesirable overt movement patterns.” [l. 17-20]

“We offer an initial reference point for therapeutic applications regarding neurorehabilitation of involuntary movements and a possible strategy to counteract the age-related increase in mirror activity.” [l. 28-30]

“Typically, stronger pMA is observed in young children and rapidly decreases as the child enters adolescence (Mayston et al., 1999). In the third decade, pMA gradually reappears (Koerte et al., 2010). This course of pMA occurrence may reflect the processes of increasing inhibition during adolescence, with the later reappearance suggesting an age-related loss of central inhibition (Carr, 2010). Thus, it has been speculated that increased levels of pMA might be one of many altered patterns of muscle activation contributing to the decline in motor performance and manual dexterity that accompanies healthy aging (Shinohara et al., 2003).” [l. 52-59]

4) When comparing EMG and EEG, did the authors take into account the approximate 25ms delay it takes neural activity to travel from the motor cortex to the peripheral muscle?

Our reply: Thank you for the comment. Our main analysis of EEG data focuses on power spectral density with rather large epochs (5 seconds). In the case of the implemented EMG analysis, we calculate mean amplitudes over the same epoch length (5 sec). In the context of these analyses the effect of neural conduction time from brain to muscle on the estimate of power can be assumed to be negligible. Furthermore, we do not directly relate EEG and EMG data in a time-locked, event-related manner. The assessment of this delay, however, would be important for the estimation of phase-phase relationship, e.g. for the corticomuscular coherence which was not the topic of the present study.

5) The authors do not mention about baseline correction for the EEG data. Was the data corrected before calculating spectral power?

Our reply: Thank you for the comment. Indeed, the EEG data was not baseline corrected before further analysis. Subtraction of a constant term, also known as baseline-correction, is important for evoked-response calculations. However, in our case we used spectral analysis and focused on frequencies > 1 Hz. Since we were not interested in the DC-term contained in the first frequency bin, baseline correction was not necessary for the estimation of power at higher frequency bands.

6) The authors should provide the script for the explanation and verbal instructions to suppress pMA.

Our reply: Thank you for this suggestion. Indeed, this valuable information was missing in our initial submission. We now provide the standardized verbal instructions in Table 1.

7) All subjects were right-handed, though they used the left-hand as the performing hand (FDIvol), with the dominant FDI probed for pMA. This is in contrast to one of the key references (Maudrich et al. 2019), in which right-handed subjects performed a similar task with their dominant right hand. I initially wondered whether there may be some asymmetry in pMA in humans in relation to hand dominance, although a brief look at the literature suggests this is not the case. Nonetheless, the authors should explain the rationale for the choice of performing hand, and address whether this is relevant in the Discussion.

Our reply: Thank you for this comment. As you already mentioned, previous investigations of right-handed subjects using prolonged isometric contractions reported no side differences in pMA between right and left-hand contractions (Addamo et al., 2009; Koerte et al., 2010; Sehm et al., 2016; Maudrich et al., 2017; Maudrich et al., 2018). Therefore, we believe it is reasonable to assume that the choice of the contracting hand in the current study, most likely did not influence the obtained result. However, we see the need to replicate our results during dominant-hand contractions in right-handed adults to prove this claim. We added this part to the Discussion.

“Investigations of right-handed adults, using similar isometric contractions to our implemented task, reported no side differences in pMA between right and left-hand contractions (Addamo et al., 2009; Koerte et al., 2010; Sehm et al., 2016; Maudrich et al., 2017; Maudrich et al., 2018). Therefore, it seems reasonable to assume that the choice of the contracting hand in the current study (left), most likely did not influence the obtained result. However, future studies are needed to replicate the voluntary inhibition of pMA during dominant hand contractions in right-handed adults.” [l. 393-399]

8) The text suggests that the onset of the target presentation in each 5s ‘trial’ served as the go cue for subjects to make their movement. Was there any measure of reaction time on each trial? It is not mentioned whether the subjects were debriefed on their self-chosen mental strategy, but one possible strategy for suppressing pMA might be to consciously make slower, more deliberate movements, which would manifest in longer RTs in the performing hand. Was this the case?

Our reply: Thank you for this comment. In order to address this question, we calculated the duration until participants reached the target field for each contraction (corresponding to 40% {plus minus} 5% MVC) i.e. ramp contraction time (RCT). Indeed, repeated measures ANOVA indicated that RCT increased from BL-block to II-block and stayed elevated thereafter compared to BL-block during III-block and IV-block. Therefore, you are correct that a possible strategy to suppress pMA might be to perform more deliberate, slower contractions. However, please note that the largest decrease in pMA amplitude was observed from BL-block to I-block where RCT only slightly increased but was not significantly different. We added a corresponding info to the Discussion.

“Continuous force data were recorded and used to 1) control that participants reached the target field and 2) compute the duration until participants reached the target field after it appeared on the computer screen for each contraction, i.e. ramp contraction time (RCT).” [l. 110-112]

“Block-wise differences in RCT were investigated using a repeated-measures ANOVA with the within-subject factor BLOCK (BL, I, II, III, IV). Bonferroni adjusted p-values of posthoc comparisons are reported within the results.” [l. 212-215]

“All participants consistently achieved the performance goal of the motor task by reaching the target field (corresponding to 40% {plus minus} 5% MVC) in every 5 s contraction during the experiment (i.e. 150 contractions). Repeated measures ANOVAs indicated a significant effect for the factor BLOCK on RCT (F(2.1, 39.9) = 11.975, p = 6.660 x 10-5, ηp² =.387). Posthoc comparison showed that RCT significantly increased from BL to II (MD = 257.098, SE = 48.409, p = 1.049 x 10-5, d = 1.188) and stayed elevated thereafter compared to BL during III (MD = 262.977, SE = 48.409, p = 6.442 x 10-6, d = 1.215) and IV (MD = 274.906, SE = 48.409, p = 1.049 x 10-5, d = 1.270).” [l. 253-259]

“Another strategy that was not reported but is manifested in increased ramp contraction times (RCT) during the experiment might be to perform more deliberate, less explosive contractions of the performing hand. Slower contractions of the performing hand, without changing the required target force level, might have allowed for easier suppression of pMA in the non-performing hand. “ [l. 414-418]

9) On a related note, it would be interesting to discuss what the authors consider to be potential strategies for inhibiting pMA (see also point 2), and the implications of this for how pMA might be reduced in pathological or higher amplitude pMA in healthy people. Given subjects were free to choose their own strategy, it seems likely that the effectiveness of different strategies could account for some of the high inter-subject variability shown in Fig. 2b.

Our reply: Thank you for this suggestion. Participants reported many different and highly individualized strategies (e.g. imagining their non-performing hand in an ice-bucket or cut-off). The main common denominator from these strategies is that all participants mentally focused on muscle relaxation in the non-performing hand. However, no clear relation between employed strategies and individual inhibition rates can be inferred from the dataset. We suggest that a promising implication for the treatment of pathological mirror movements/ pMA increase in the elderly might be an EMG-feedback-based approach as part of the Discussion.

“After the experiment, participants were asked to describe their self-chosen strategy used to inhibit pMA. While many different and highly individualized strategies have been mentioned, a main common denominator can be inferred. All participants, in one way or another, mentally focused on non-activation of the non-performing hand during each contraction of the performing hand. Thus, constantly paying attention to muscle relaxation in non-performing limbs might be a prerequisite for successful pMA inhibition besides being aware of its presence. However, no clear relation between individually employed strategies and participants inhibition rates is recognizable so that a decisive conclusion cannot be drawn from this dataset.” [l. 406-414]

“One possible intervention might be to implement online feedback of involuntary muscular activity to voluntarily decrease the level of EMG and even possibly the occurrence of overt MM, as it has been demonstrated previously in brain-injured young adults (Lazarus, 1992). However, the transfer of pMA-mechanisms to pathological MM remains elusive and requires further research. Nevertheless, this inhibitory feedback-based approach appears additionally promising in the context of counteracting the age-related increases in pMA, which might help prevent the decline in motor performance documented in the elderly.” [l. 507-513]

10) Line 110: “during which muscles should be completely relaxed”. The authors perform some analysis of non-task related epochs for the EEG data, but did they confirm from the actual data that subject EMG (in both hands) was consistently absent during these periods as well, and that there were no systematic (e.g. fatigue/attention-related) changes over the course of the experiment?

Our response: Thank you for this comment. We additionally computed mean EMG amplitudes of pre-contraction epochs (4s before the onset of each contraction) and compared them across blocks to address this concern. Furthermore, we visually inspected and confirmed the absence of overt muscle activity in these epochs. Mean EMG amplitudes of FDIVol showed no change over the course of the experiment, confirming the absence of systematic baseline changes. However, corresponding to contraction epochs of FDIpMA, mean EMG amplitudes in pre-contraction epochs of FDIpMA were significantly reduced in all blocks where participants were informed about the nature of the experiment (except for IV-block). This effect of baseline reduction of FDIpMA is most likely the consequence of the inherent performance goal of the experiment: to inhibit muscle activity in FDIpMA, which is introduced following the BL-block.

The respective part of the Methods and Results now reads as follows:

“Mean EMG amplitudes were additionally estimated for pre contraction epochs (4 s rest periods between consecutive contractions) for FDIVol, as well as FDIpMA. The absence of overt muscle activity in these epochs was checked and confirmed visually, i.e. the relaxation of participants between each consecutive contraction. This control condition was implemented to test for systematic fatigue- or attention-related changes in baseline EMG amplitudes across the experiment.” [l. 169-174]

“For FDIVol and FDIpMA (contraction & pre-contraction epochs), block-wise changes in amplitude were investigated using separate repeated-measures ANOVAs with the within-subject factor BLOCK (BL, I, II, III, IV). Here, the significance level was Bonferroni adjusted for 4 pairwise posthoc comparisons (BL vs. I, BL vs. II, BL vs. III, BL vs. IV; padj < 0.0125).” [l. 215-219]

“Mean EMG amplitudes of FDIVol during pre-contraction epochs showed no difference between blocks, suggesting the absence of systematic changes in EMG baseline over the course of the experiment (F(2.954, 56.120) = 1.615, p = 0.179, ηp² =.078). In case of FDIpMA pre-contraction epochs, a significant effect of BLOCK was found (F(2.286, 43.440) = 5.327, p = 0.006, ηp² =.219). Corresponding to contraction epochs of FDIpMA, mean EMG amplitudes were significantly reduced in all blocks where participants were informed about the nature of the experiment (except for IV-block) compared to BL: BL to I (MD = -0.003, SE = 8.368 x 10-6, p = 0.006, d = -.802), BL to II (MD = -0.003, SE = 8.368 x 10-6, p = 0.002, d = -.865) & BL to III (MD = -0.003, SE = 8.368 x 10-6, p = 0.003, d = -.855).” [l. 270-278]

11) The stated goal of the task is to minimize pMA in the non-performing hand while maintaining consistent FDIvol EMG (L118-119). This seems to be a critical point - any concomitant reductions in FDIvol EMG would suggest subjects can inhibit pMA simply by reducing overall motor drive, rather than engaging any specific inhibition of the non-performing hand. In actual fact, the authors report increases in FDIvol during III and IV, in contrast to the decreases in pMA. This is suggested in the discussion to be due to fatigue effects as shown previously but is somewhat conflicting with the goal stated in the introduction. Overall, the details and presented data on FDIvol data were lacking. It would be important to know that subjects were indeed consistently achieving the desired force level with the performing hand in order to rule out changes here as a confound. Was there any measure of subject performance in the vertical cursor target task itself? And were there any within-subject relationships in changes in FDIvol and FDIpma amplitudes over the course of the experiment?

Our reply: Thank you for your comments. The goal of our task was to reduce pMA while maintaining constant motor performance, not constant FDIvol EMG: “The goal was to minimize EMG amplitude of the non-performing hand without changing the motor performance of the left hand.” Successful task performance was achieved by reaching the target field (corresponding to 40% {plus minus} 5% MVC) in each of the 150 trials throughout the experiment. Factually, all participants reached the desired target field in 100% of cases (150 contractions throughout the experiment), which was confirmed through examination of continuous force data. Therefore, we are confident that suppression of pMA was not confounded by overall decreased motor drive of the contracting hand.

On the other hand, EMG amplitudes of FDIvol were not required to stay constant and expected to change over the course of the experiment. We initially interpreted the slight increase in FDIvol EMG being due to fatigue effects over the course of the experiment, however without formally testing for it. To actually verify this point, we estimated the median frequency (MDF) of the power spectra from all voluntary contractions (Phinyomark et al., 2012; Maudrich et al., 2019) and compared them block-wise by a repeated measures ANOVA. We found no significant change in MDF between blocks (F(2.061, 39.168) = 0.173, p = .848, ηp² =.009). The stability of estimated MDF over the course of the experiment (please see figure S1 below) does not support peripheral fatigue in FDIvol. We therefore removed this interpretation from the manuscript. Alternatively, this slight increase in FDIvol amplitude could be also due to increased sensory afferent drive (Sinkjaer et al., 2000). It seems reasonable to assume that continuous performance of 150 contraction would lead to some sort of discomfort in the contracting fingers of participants, most likely leading to elevated sensory feedback. Actually, slight peripheral discomfort was reported by some of our participants after the experiment. Additionally, we cannot confirm any systematic within-subject relationships in changes between FDIvol and FDIpma amplitudes over the course of the experiment (all p > 0.05 after Bonferroni correction). Following your suggestion, we now present FDIvol EMG data as part of Figure 2 (2 A).

Figure S1. Supplemental figure showing block-wise median frequency (MDF) of FDIvol.

12) The authors suggest that subjects with minimal changes in pMA over the course of the task may have been due to floor effects, since pMA is already a fraction of MVC amplitudes at BL (L335-336). It cannot be inferred from Fig. 2 whether this is indeed the case - were the subjects in 2b with minimal changes also the ones with low BL pMA amplitude (in 2a)? It should be fairly straightforward to check this within the data in order to support the authors’ suggestion.

Our response: Thank you for this suggestion. Indeed, we found a strong negative correlation between initial levels of pMA (pMA amplitude in BL-block) and the percentage change in pMA amplitude from BL-block to I-block (r(20) = -.813, p = 1.32 × 10-5). This suggests that participants with higher initial pMA amplitudes showed the highest rates of inhibition from BL-block to I-block. However, as discussed in the manuscript, this observed correlation must be interpreted with caution. It might be possible that the floor-effect of EMG baseline amplitude (signal-to-noise ratio) could be limiting the potential for inhibition of participants with initially low pMA amplitudes. The respective part of the Discussion now reads as follows:

“Interestingly, we observed different rates of inhibition between participants (see Figure 2 C). In conjunction with this, we found a strong negative correlation between initial levels of pMA (pMA amplitude in I-block) and the percentage change in pMA amplitude from BL-block to I-block (see Figure 2 D). This suggests that participants with higher initial pMA amplitudes showed the highest rates of inhibition. However, this correlation must be interpreted with caution. In some participants, very low initial pMA values and inhibition rates were present, suggesting on the one hand that pMA cannot demonstrate a strong decrease due to the “floor effect”. On the other hand, it might be possible that the signal-to-noise ratio of the surface EMG recordings could be limiting the potential for inhibition of participants with initially low pMA amplitudes in the present experiment. Intramuscular needle EMG might be better suited to detect the most subtle involuntary discharges of motor units and could, therefore, be used in future studies to resolve the question of whether pMA can be completely eliminated. “ [l. 380-392]

13) In the correlation analysis (Fig. 4), although the BL block is not significant and the I block is, this does not strictly support the conclusion that the inverse correlation emerges in the I block, given how similar the Pearson correlation values are. Block III and IV do show progressively stronger correlations, although here, the effect of the outlier at -3, +4 (presumably the same subject in all panels) is concerning. A more convincing demonstration of a genuinely strengthening correlation across blocks would require direct comparisons between blocks (e.g. Steiger’s Z test), as well as an estimation method robust to outliers (e.g. bootstrapping).

Our reply: Thank you for this comment. We agree that the outlier may appear to be driving the effect but decided not to exclude this participant as we do believe that the observed pMA is a true value and not the effect of measurement errors or artifacts. However, in order to address this concern, we implemented a more robust estimation method. First, we confirmed that the residuals of the linear regression between frontal delta power and pMA values were normally distributed by Lilliefors-testing for each block separately. By confirming the normal distribution of the residuals, we justify the usage of Pearson’s linear correlation (Casson and Farmer, 2014). Secondly, we estimated the significance of Pearson’s correlation coefficients by permutation testing (1000 permutations) (Manly, 2006). The Pearson correlation coefficients were considered to be significant when they eclipsed the 95th percentile of permuted data. We now report family-wise error corrected p-values for all pairwise correlations and show them as part of Figure 4. Furthermore, we compared the significant correlation coefficients during the I-block with the IV-block to test for a “strengthening” of the association between pMA and frontal delta power over the course of the experiment. Therefore, we employed Fisher’s r to z-transformation and tested for a significant difference in the correlation coefficients. This comparison was not significant (z-score = 1.139, p = 0.127). We therefore removed the phrasing “the correlation strengthened over the course of the experiment” as well as the interpretation thereof from the manuscript as we cannot claim a true difference in correlation strength based on our dataset.

“To test our hypothesis that the amplitude of pMA observed during naïve (BL) and informed blocks (I, II, III, IV) is inversely associated with the individual delta power in the frontal ROI for each respective block, linear regression analyses were carried out on all data per block. Delta power served as dependent variables and corresponding pMA amplitudes served as predictors. Next, we assessed normality of residuals by way of Lilliefors testing (α = 0.05). All residuals were normally distributed. Thereafter, Pearson correlation coefficients were estimated for each block. To evaluate the significance of our results, delta power and pMA amplitude vectors were randomly permuted, resulting in a distribution of Pearson correlation coefficients from 1000 permutations (Manly, 2006). The Pearson correlation coefficients were deemed significant when they eclipsed the 95th percentile of permuted data. Family-wise-error corrected p-values of Pearson correlation coefficients are reported with the results. Additionally, Fisher’s r to z-transformation was implemented to test for a significant difference in the correlation coefficients of I-block and IV-block (Fisher, 1921).” [l. 232-244]

14) The authors suggest that the increasingly negative correlation between pMA and log delta power, particularly in III and IV blocks, is due to fatigue and “increased inhibitory demands” (L363-367). The slight increases in pMA between blocks I and IV in Fig. 2 might support this indirectly, but doesn’t this interpretation suggest that larger increases in delta power should also occur in order to maintain pMA suppression as the task progresses? On the other hand, the results presented in Figs. 2 and 3 suggest that the largest delta power increase, and to some extent, the strongest pMA inhibition, both occur between the BL and I block. It is difficult to reconcile these points. At the least, it would help for the authors to present comparisons between BL and the other ‘informed’ blocks as well in Fig. 3. Also, have the authors considered a correlation analysis using the differences between blocks, rather than the individual block values themselves? This may provide better support for a stricter “within-subject” interpretation regarding increasing fatigue levels.

Our response: Thank you for your considerations. In the initial version of our manuscript we reported an increasingly negative correlation over the course of the experiment solely based on the descriptive comparison of correlation coefficients. However, as pointed out in comment 13 (please see above), a convincing demonstration of a genuinely strengthening correlation across blocks would require direct statistical comparisons between blocks. Therefore, we employed Fisher’s r to z-transformation and tested for a significant difference in the correlation coefficients obtained during I-block and IV-block. This comparison was not significant (z-score = 1.139, p = 0.127). We therefore removed the phrasing “the correlation strengthened over the course of the experiment” as well as the interpretation thereof from the manuscript as we cannot claim a true difference in correlation strength based on our dataset.

Nevertheless, we agree that the presentation of delta power of all informed blocks would improve the manuscript and thus added this information to Figure 3.

15) Line 383: The authors speculate that the reason they did not find effects in the theta band may have been due to task differences from previous studies related to the duration/frequency of contraction. Could the authors expand on why they think these task differences might influence theta power?

Our response: Thank you for this comment. After giving this issue more thought, we now believe that the lack of effects in theta band is related to the absence of online feedback during motor performance and not necessarily due to differences in duration/frequency of the motor tasks. Increases in theta power were often demonstrated during voluntary response inhibition paradigms (Cohen et al., 2011; Andreou et al., 2017). In this context, voluntary response inhibition is closely related to feedback on task performance, meaning that inhibitory strategies and their success rates can be assessed and optimized during task performance (Yamanaka and Yamamoto, 2010). Thus, this ability to strategically fine-tune inhibitory strategies (Kirmizi-Alsan et al., 2006; Yamanaka and Yamamoto, 2010; Tisseyre et al., 2019) is a major difference compared to the task we employed in this study. In the present study participants were unaware of their task performance (inhibition of pMA) during the contraction blocks. Therefore, strategic fine-tuning is unlikely to have occurred due to participants’ uncertainty regarding successful inhibition.

The respective part of the Discussion now reads as follows:

“Contrary to our hypothesis, we did not observe changes in theta power from BL-block to I-block. This seems surprising, as a previous investigation found a theta power increase associated with lateralized inhibition of symmetrical movements in the fronto-mesial area, attributed to executive processes (Tisseyre et al., 2019). This contradiction may be explained by differences between our task and those from previous studies showing theta power increases during motor inhibition. Such increases in theta power were often demonstrated during voluntary response inhibition paradigms (Cohen et al., 2011; Andreou et al., 2017). In this context, voluntary response inhibition is closely related to feedback on task performance, meaning that inhibitory strategies and their success rates can be assessed and optimized during task performance (Yamanaka and Yamamoto, 2010). Thus, this ability to strategically fine-tune inhibitory strategies (Kirmizi-Alsan et al., 2006; Yamanaka and Yamamoto, 2010; Tisseyre et al., 2019) is a major difference compared to the task we employed in this study. Due to the inherent characteristics of the motor task in the present study, where participants were entirely unaware regarding successful inhibition of pMA throughout the contraction blocks, strategic fine-tuning is unlikely to have occurred due to participants’ uncertainty regarding successful inhibition. This mechanistic difference may serve as an explanation for the absence of an increase in theta power as participants could not have been certain regarding successful pMA inhibition and, therefore, stable theta dynamics, reflecting voluntary inhibitory response mechanisms did not emerge.” [l. 454-472]

16) Line 402: The authors note a limitation of their study in dissociating the effects on pMA suppression of directed attention vs inhibitory processes. This is an inevitable consequence of the task design where subjects are told about pMA and encouraged to inhibit it, and there is no control group. While I agree with the authors that EMG measurements are a useful technical advance, it is not clear what kind of control or alternative tests they have in mind here to dissociate the two processes they refer to, other than perhaps a similar protocol to the cited Addamo et al. 2010 paper.

Our response: You are right. We failed to mention that in order to dissociate directed attention and inhibitory control, one has to strictly separate these mechanisms for example by employing distinct intervention groups. Following this line of thought, we suggest that a between-subject design, incorporating two distinct intervention groups and one control group might be an alternative. One intervention group would be informed about pMA presence but instructed to ignore it, while the other intervention group is additionally instructed to actively inhibit pMA. The control group would be naïve regarding pMA throughout the experiment. This is in contrast to the within-subject protocol used by Addamo et al. 2010, where the same group of participants was informed about pMA but asked to ignore it and thereafter instructed to actively inhibit pMA. We expanded on this point in the Discussion.

“As a limitation of the present study, we are not able to disentangle the distinct effects of directed focus on pMA presence and actual inhibitory processes on the observed behavioral suppression of pMA. One previous study observed that pMA remained unchanged after young adults were informed of it but asked to ignore their motor overflow. Only when participants were requested to inhibit involuntary co-activation with and without visual feedback were they able to reduce pMA (Addamo et al., 2010). While this within-subject study protocol seems appropriate to disentangle the effects of directed focus and actual inhibitory processes on pMA suppression in general, we suggest that a between-subject protocol might be an alternative. As part of this between-subject protocol, one intervention group would be informed about pMA presence but instructed to ignore it, while a separate intervention group is additionally instructed to actively inhibit pMA. A control group would be naïve regarding pMA throughout the experiment. This experimental design could be repeated over several days to investigate the stability of these mechanisms or even learning effects in pMA inhibition over time. However, as stated before, force measurements are insufficient in capturing neurophysiological aspects of pMA in its entirety, rendering future studies incorporating EMG measurements necessary to answer these questions raised above. “ [l. 486-501]

Minor

17) Please spell out all acronyms again in the Discussion in order not to tax the reader’s working memory or cause them to move back and forth.

Our reply: Thank you for this comment. All acronyms have been spelled out again in the Discussion.

18) Line 36: there is no need to introduce the acronym MM here, as it is not used again in the Introduction.

Our reply: Thank you for pointing this out. We removed this acronym from the Introduction. We introduce this acronym now as part of the Discussion.

19) Figure 1 seems to serve primarily as a methods figure for the EEG electrode and ROI configuration, but also includes EEG results, which are shown in a different form in Figure 3. The rationale for splitting it this way is not clear. As a reader, an alternative figure 1 depicting the task design (perhaps showing some FDIvol/FDIpma activity) would have been helpful.

Our reply: Thank you for this suggestion. Indeed, we already display EEG results in Figure 3. Accordingly, we adapted Figure 1 to depict the task design and exemplary EMG traces to better serve as a methods figure.

20) Line 121: “Online feedback was not provided.” Does this mean that subjects were not able to see a display showing EMG in their FDI muscles, but were able to see both hands at all times?

Our reply: You are correct. We clarified this point in the manuscript.

“That means that participants neither received online EMG-feedback nor verbal instructions during contractions. Participants could see both hands at all times but were instructed to fixate their gaze on the computer screen in front of them during all contractions.” [l. 124-126]

21) Lines 147, 201, 220, 234...: “analyses“

Our reply: Thank you for noticing. We corrected all instances of “analyzes”.

22) Lines 202-210: There seems to be some unnecessary repetition here. Excepting the GG correction, the analyses for the two FDIs appear identical.

Our response: You are right, thank you for noticing. We deleted the unnecessary repetition. This part now reads as follows:

“For FDIVol and FDIpMA (contraction & pre-contraction epochs), block-wise changes in amplitude were investigated using separate repeated-measures ANOVAs with the within-subject factor BLOCK (BL, I, II, III, IV). Here, the significance level was Bonferroni adjusted for 4 pairwise posthoc comparisons (BL vs. I, BL vs. II, BL vs. III, BL vs. IV; padj < 0.0125). The same model was implemented to test for block-wise changes in latency of FDIpMA. In the case of a violation of the sphericity assumption, Greenhouse-Geisser correction was implemented.” [l. 215-221]

23) The authors have used the abbreviations BL and I in the figures. It would be clearer to add definitions as well.

Our reply: Thank you for this suggestion. We added definitions to all abbreviations in all figures.

24) In figure 3B, could the authors add units to the color bar?

Our reply: Thank you for noticing. We added the unit to the color bar (%).

References

Addamo PK, Farrow M, Hoy KE, Bradshaw JL, Georgiou-Karistianis N (2009) The influence of task characteristics on younger and older adult motor overflow. Q J Exp Psychol 62:239-247.

Addamo PK, Farrow M, Bradshaw JL, Moss S, Georgiou-Karistianis N (2010) The effect of attending to motor overflow on its voluntary inhibition in young and older adults. Brain and cognition 74:358-364.

Andreou C, Frielinghaus H, Rauh J, Mussmann M, Vauth S, Braun P, Leicht G, Mulert C (2017) Theta and high-beta networks for feedback processing: a simultaneous EEG-fMRI study in healthy male subjects. Transl Psychiatry 7:e1016.

Carr LJ (2010) Some reflections on mirror movements. Dev Med Child Neurol 52:1077-1078.

Casson RJ, Farmer LD (2014) Understanding and checking the assumptions of linear regression: a primer for medical researchers. Clinical & Experimental Ophthalmology 42:590-596.

Cohen MX, Wilmes K, Vijver I (2011) Cortical electrophysiological network dynamics of feedback learning. Trends in cognitive sciences 15:558-566.

Fisher RA (1921) On the” Probable Error” of a Coefficient of Correlation Deduced from a Small Sample. Metron 1:3-32.

Kirmizi-Alsan E, Bayraktaroglu Z, Gurvit H, Keskin YH, Emre M, Demiralp T (2006) Comparative analysis of event-related potentials during Go/NoGo and CPT: decomposition of electrophysiological markers of response inhibition and sustained attention. Brain Res 1104:114-128.

Koerte I, Eftimov L, Laubender RP, Esslinger O, Schroeder AS, Ertl-Wagner B, Wahllaender-Danek U, Heinen F, Danek A (2010) Mirror movements in healthy humans across the lifespan: effects of development and ageing. Dev Med Child Neurol 52:1106-1112.

Lazarus JC (1992) Associated movement in hemiplegia: the effects of force exerted, limb usage and inhibitory training. Archives of physical medicine and rehabilitation 73:1044-1049.

Manly BF (2006) Randomization, bootstrap and Monte Carlo methods in biology: CRC press.

Maudrich T, Kenville R, Lepsien J, Villringer A, Ragert P (2018) Structural Neural Correlates of Physiological Mirror Activity During Isometric Contractions of Non-Dominant Hand Muscles. Scientific Reports 8:9178.

Maudrich T, Kenville R, Lepsien J, Villringer A, Ragert P, Steele CJ (2017) Mirror Electromyografic Activity in the Upper and Lower Extremity: A Comparison between Endurance Athletes and Non-Athletes. Front Hum Neurosci 11:485.

Maudrich T, Kenville R, Nikulin VV, Maudrich D, Villringer A, Ragert P (2019) Inverse relationship between amplitude and latency of physiological mirror activity during repetitive isometric contractions. Neuroscience 406:300-313.

Mayston MJ, Harrison LM, Stephens JA (1999) A neurophysiological study of mirror movements in adults and children. Ann Neurol 45:583-594.

Phinyomark A, Thongpanja S, Hu H, Phukpattaranont P, Limsakul C (2012) The Usefulness of Mean and Median Frequencies in Electromyography Analysis. In: Computational Intelligence in Electromyography Analysis (Naik GR, ed). Rijeka: IntechOpen.

Sehm B, Steele CJ, Villringer A, Ragert P (2016) Mirror Motor Activity During Right-Hand Contractions and Its Relation to White Matter in the Posterior Midbody of the Corpus Callosum. Cereb Cortex 26:4347-4355.

Shinohara M, Keenan KG, Enoka RM (2003) Contralateral activity in a homologous hand muscle during voluntary contractions is greater in old adults. Journal of applied physiology (Bethesda, Md : 1985) 94:966-974.

Sinkjaer T, Andersen JB, Ladouceur M, Christensen LO, Nielsen JB (2000) Major role for sensory feedback in soleus EMG activity in the stance phase of walking in man. The Journal of physiology 523 Pt 3:817-827.

Tisseyre J, Marquet-Doleac J, Barral J, Amarantini D, Tallet J (2019) Lateralized inhibition of symmetric contractions is associated with motor, attentional and executive processes. Behavioural brain research 361:65-73.

Yamanaka K, Yamamoto Y (2010) Single-trial EEG power and phase dynamics associated with voluntary response inhibition. J Cogn Neurosci 22:714-727.
